# Laparoscopic excision of a solitary fibrous tumor originating from the abdominal wall: a case report

**DOI:** 10.1093/jscr/rjaa602

**Published:** 2021-03-15

**Authors:** Kouki Imaoka, Masahiro Nishihara, Megumi Yamaguchi, Yukari Kawasaki, Keizo Sugino

**Affiliations:** Department of Surgery, Akane-Foundation, Tsuchiya General Hospital, Hiroshima, Japan; Department of Surgery, Akane-Foundation, Tsuchiya General Hospital, Hiroshima, Japan; Department of Surgery, Akane-Foundation, Tsuchiya General Hospital, Hiroshima, Japan; Department of Surgery, Akane-Foundation, Tsuchiya General Hospital, Hiroshima, Japan

## Abstract

Solitary fibrous tumors (SFTs) are mesenchymal fibroblastic tumors, and forms of SFTs that originate from the abdominal wall are extremely rare. Here we report a case of a nonpalpable SFT along the abdominal wall. Abdominal magnetic resonance (MR) imaging showed a well-circumscribed mass measuring 5 cm in diameter with heterogeneous signal intensity on T2-weighted MR images; this mass was diagnosed as a benign abdominal tumor of unknown origin. Successful laparoscopic excision of the tumor was performed. Histological examination revealed a benign extrapleural SFT. No tumor recurrence was observed after 20-month follow-up. This is the first case of laparoscopic excision of an SFT originating from the abdominal wall. Our report highlights the safety and usefulness of laparoscopic excision of abdominal wall tumors such as SFTs. This approach is an underutilized surgical treatment that can be applied to select cases of SFT in the abdominal cavity.

## INTRODUCTION

Solitary fibrous tumors (SFTs) are rare slow-growing fibrous tumors of mesenchymal origin that usually arise from the pleura [1]. Recently, SFTs have been recognized to occur in any part of the body, especially in the abdominopelvic region, most commonly in the retroperitoneum [2, 3]. However, SFTs originating from the abdominal wall are extremely rare [1, 4–6]. Preoperative diagnosis of SFTs is difficult because of associated nonspecific symptoms, laboratory values and imaging features; thus, surgical resection followed by pathological evaluation is warranted for diagnosing SFTs [7]. Compared with SFTs of the pleura, SFTs in the abdominopelvic region are often larger with a higher malignant potential at the time of detection [2]. Therefore, SFT excision is commonly performed via the open technique, and laparoscopic excision is rare. Here we report a case of an SFT originating from the abdominal wall, which was successfully excised laparoscopically. This is the first case of laparoscopic excision of an SFT originating from the abdominal wall.

## CASE REPORT

**Figure 1:**
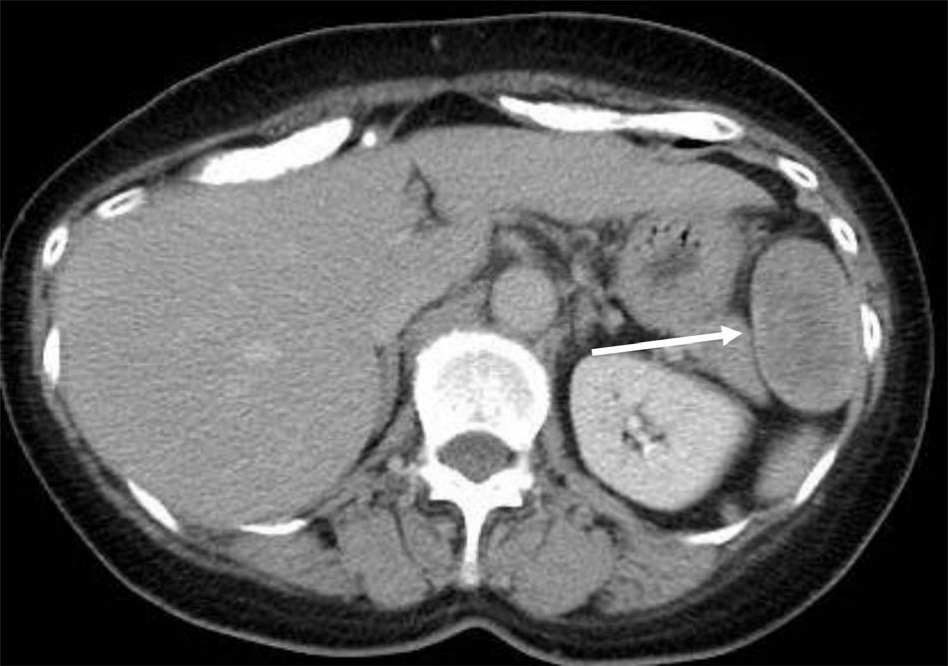
Radiological findings. Computed tomography showing a well-circumscribed soft tissue mass measuring 5.1 × 3.2 × 2.6 cm in the left anterolateral abdominal wall (white arrow).

**Figure 2:**
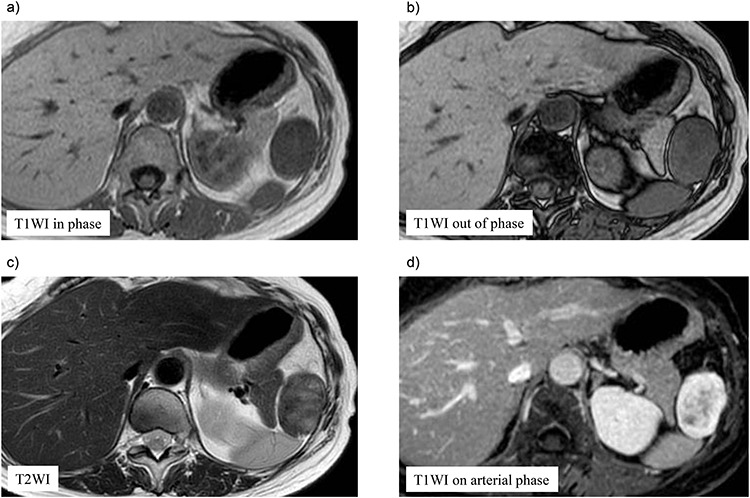
Preoperative magnetic resonance (MR) imaging findings. The tumor shows low signal intense mass on T1-weighted MR images in phase (**a**) and out of phase (**b**) and a heterogeneous hyperintense mass on T2-weighted MR images (**c**). (**d**) The tumor shows hypointense tumor on T1-weighted MR images with delayed enhancement in the arterial phase.

A 70-year-old woman underwent right thyroidectomy for thyroid cancer 7 years ago. An abdominal tumor was incidentally detected on computed tomography (CT) during a follow-up visit, and the patient was referred to our hospital for further evaluation. On physical examination, the mass was nonpalpable and nontender. Laboratory test results were nonspecific. Abdominal contrast-enhanced CT revealed a well-circumscribed soft tissue mass measuring 5.1 × 3.2 × 2.6 cm at the left anterolateral abdominal wall (Fig. 1). There were no signs of invasion of the adjacent organs. Magnetic resonance imaging (MRI) revealed a hypointense tumor on T1-weighted MR images with delayed enhancement in the arterial phase and a heterogeneous hyperintense tumor on T2-weighted MR images (Fig. 2a–d). The tumor was preoperatively diagnosed as a gastrointestinal tumor or a benign tumor, and it was mainly supplied by abdominal wall arteries.

**Figure 3:**
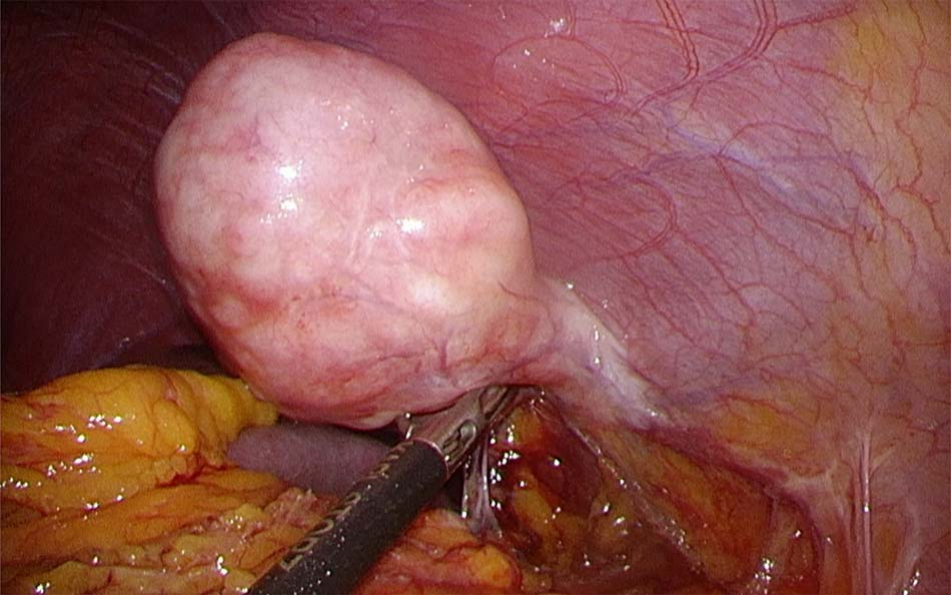
Intraoperative findings. Laparoscopic view showing the solitary fibrous tumor bulging into the abdominal cavity with a stalk, with its peritoneal surface covered with hard nodules.

**Figure 4:**
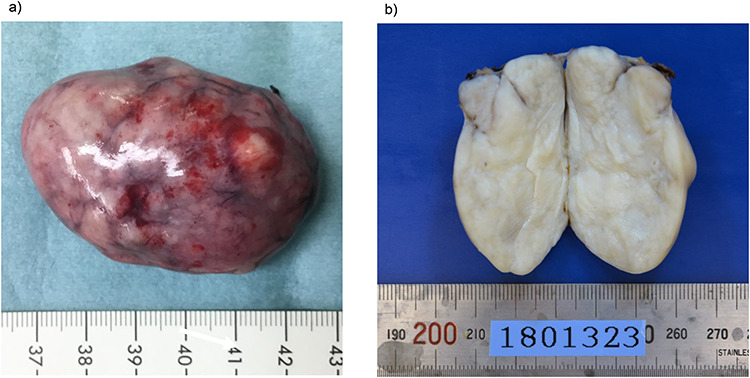
Macroscopic findings of the resected specimen. (**a**) The resected specimen measuring 5.9 × 3.5 cm has a multinodular whitish appearance. (**b**) The cut surface of the resected tumor is solid and yellowish-white.

The mass was excised using a five-trocar approach. We separated the omentum from the left upper abdominal wall and

found a pedunculated mass on the anterior abdominal wall that had a stalk and bulged into the abdominal cavity, with its peritoneal surface covered with hard nodules (Fig. 3). The tumor was excised from the peritoneum, along with parts of the inner layer of the transversus abdominis muscle and was removed via the umbilicus. The abdomen was reinsufflated, and on evaluating the excision site, prosthetic mesh reinforcement of the abdominal wall was not required. The operative duration was 92 min, and the intraoperative blood loss was 5 ml. The resected tumor measured 5.9 × 3.5 cm in size and had a multinodular whitish appearance with a sufficient surgical margin (Fig. 4a, b).

**Figure 5:**
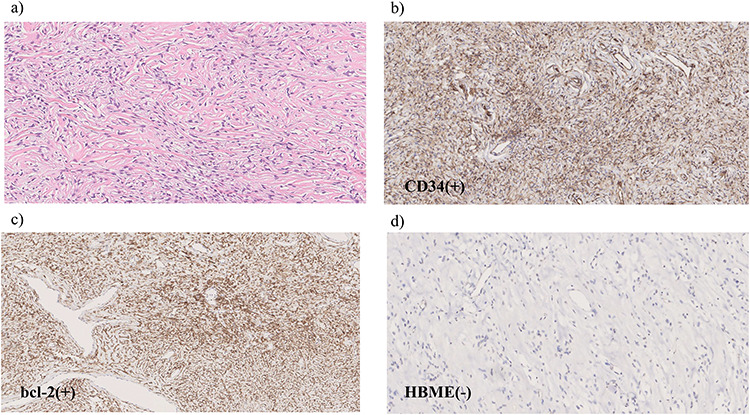
Microscopic findings of the specimen. (**a**) Hematoxylin and eosin staining reveals spindle-shaped tumor cells with elongated nuclei. Immunohistochemical examination shows tumor cells positive for CD34 (**b**) and Bcl-2 (**c**) but negative for HBME-1 (**d**).

Hematoxylin and eosin staining revealed spindle-shaped tumor cells with elongated nuclei (Fig. 5a). Immunohistochemical examination showed that the tumor cells were positive for CD34 and Bcl-2 but negative for HBME-1 (Fig. 5b–d). The tumor cells showed no malignant features, such as high mitosis, pleomorphism, hemorrhage and necrosis. Based on these findings, we diagnosed the tumor as a benign extrapleural SFT.

The patient was discharged without complications on postoperative Day 4. No tumor recurrence was confirmed after 20 months of postsurgical follow-up.

## DISCUSSION

SFTs originating from the abdominal wall are extremely rare, and only 16 cases have been reported in English literatures [1, 4–6].

The clinical presentation of extrapleural SFTs depends on both tumor localization and size, mostly caused by the compression of adjacent structures.

Radiological diagnosis of SFTs is usually very difficult because SFTs do not have typical imaging features; hence, histopathological examination is required. SFTs are composed of spindle cells with varying amounts of hyalinized collagen, usually arranged in a ‘pattern-less pattern’ and with heterogeneous distribution of cellular density. This is why fine-needle aspiration biopsies rarely provide sufficient tissue for a definitive diagnosis. Immunohistochemical staining may be used to accurately diagnose the tumor; immunohistochemical markers, such as CD34, CD99, BCL-2, are commonly used to characterize SFTs. SFTs are positive for CD34 and BCL-2 in 78%–100% and 96% of cases, respectively [7].

The most effective treatment for SFTs is surgical excision with a sufficient surgical margin. Malignant lesions are distinguished from benign lesions based on high cellularity of >4 mitotic figures per 10 high-power fields, pleomorphism, hemorrhage and the presence of necrosis [8]. SFTs are frequently found to be benign, although some cases of malignant SFTs have been reported, with a 10.8% recurrence rate [9]. Extrathoracic SFTs located in the abdomen, pelvis or retroperitoneum tend to behave more aggressively than thoracic SFTs [2]. A retrospective analysis suggested that a positive resection margin was significantly correlated with local recurrence, while tumor size >10 cm and high mitosis rate were significantly correlated with a higher incidence of metastases and higher mortality rate [3]. However, clinical behavior cannot always be predicted based on histological features. Recurrence or metastasis has been noted in histologically benign SFTs; thus, strict follow-up practices are recommended [9].

It is generally technically difficult to excise large abdominopelvic tumors because poor visualization of the operative field may cause massive intraoperative bleeding.

In the present case, the tumor was incidentally detected before the onset of any symptoms. We selected a laparoscopic approach because of the tumor location at the anterior abdominal wall, which could provide us good surgical visualization. Indeed, we confirmed the stalk of the tumor and excised it safely and easily with a sufficient surgical margin via laparoscopic surgery. This is the first case of laparoscopic excision of SFT originating from the abdominal wall. The laparoscopic approach prevented abdominal wall trauma associated with open surgery in addition to providing the well-known benefits of faster recovery and better cosmesis. It also prevented excision of the oblique abdominal wall muscle, thus averting abdominal wall reconstruction using a prosthetic mesh.

In summary, our report highlights the safety and usefulness of laparoscopic excision of abdominal wall tumors, particularly pedunculated tumors, such as SFTs, bulging into the abdominal cavity. This is an underutilized surgical treatment that can be applied to select cases of SFTs in the abdominal cavity.

## CONFLICT OF INTEREST STATEMENT

None declared.

## INFORMED CONSENT

Informed consent was obtained from the patient for whom identifying information is included in this article.
